# Supplementary material for: Citizen science with colour blindness: A case study on the Forel-Ule scale
Source: PLoS One. 2021 Apr 19;16(4):e0249755. doi: 10.1371/journal.pone.0249755 (PMC8055037; doi:10.1371/journal.pone.0249755)
Supplement: Attachment — Submitted filename: Response to Reviewers.pdf [file pone.0249755.s001.pdf]

Dear editor,

We thank the academic editor and referee for their swift and constructive reviews. A point-by-point response is included below, with the referee's comments in italic text and our response in normal text. An edited version of the manuscript, with changes indicated, has been uploaded. The editorial comments have also been incorporated in this revised manuscript.

On behalf of the authors,
Kind regards,
Olivier Burggraaff

Referee #1
1. Subsection 2.2 presents details of the calculations necessary to simulate the effect of the various colour deficiency conditions on the discriminability of the samples present in the FU scale. However, readers can benefit from an early introduction to the rationale of this mapping perhaps in the Introduction section or early in section 2.2. This can be done by discussing the methods by Brettel et al (1997) on which the simulations are based.

The start of Section 2.2 has been expanded to better and more thoroughly explain the principles the simulations are based on. This should make the manuscript more accessible and interesting to the broad audience of PLOS ONE.

2.The authors propose modifying current data entry protocols to include measurements of uncertainty associated with reference similarity for normal and deficient colour vision (lines 217-220). This is a very interesting idea and useful conclusion of the paper. I recommend to expand on this idea based on the presented results. By doing that this manuscript will became a very useful tool for designing new protocols improving not only data collected but also its efficiency,

The referee is right to point out that a more thorough discussion on this topic would make the manuscript more useful to readers. Thus we have expanded this section of the discussion significantly to include three concrete suggestions as well as relevant references. A summary of these methods has also been included in the Conclusions & recommendations section.

Editorial comments
The authors' affiliations have been edited to fit the required format.

Several acknowledgements have been added that were pending approval from the relevant people.

Corrected one reference (Novoa et al. 2014) to include the full name of the journal.
